# Pilot Data Suggest That Obesity and Presence of Malignancy Are Associated with Altered Immune Cell Infiltration in Endometrial Biopsies

**DOI:** 10.3390/jcm13237248

**Published:** 2024-11-28

**Authors:** Eline Jacques, Anouk van den Bosch, Peggy de Vos van Steenwijk, Loes Kooreman, Bert Delvoux, Andrea Romano, Henrica Werner

**Affiliations:** 1 Department of Obstetrics and Gynecology, Maastricht University Medical Centre, 6229 HX Maastricht, The Netherlands; 2 GROW-Research Institute for Oncology and Reproduction, Maastricht University, 6229 ER Maastricht, The Netherlands; 3 Department of Pathology, Maastricht University Medical Centre, 6229 HX Maastricht, The Netherlands

**Keywords:** endometrial cancer, endometrial biopsy, inflammation, immunity, obesity, body mass index

## Abstract

(1) **Background**: The worldwide endometrial cancer (EC) incidence is rising, amongst others linked to obesity, type 2 diabetes mellitus (T2DM), and metabolic syndrome, possibly due to low-grade adipose tissue inflammation. We studied immune cell infiltration in the endometrium in relation to diagnosis and obesity. (2) **Methods**: A cohort was created (*n* = 44) from postmenopausal women, lean (*n* = 15) and obese (*n* = 29), with bleeding complaints due to EC (*n* = 18) or benign pathology (*n* = 26). Endometrial biopsies were used to study the immune microenvironment and stained for macrophages (CD68 and CD163), T-cells (CD3 and CD8), and NK-cells (CD56). (3) **Results**: Malignant samples showed reduced intraepithelial CD3+ and CD8+ T-cells and increased stromal CD3+ T-cells. In obese patients, increased intraepithelial CD3+ and CD8+ T-cells were detected, especially in obese patients with T2DM. Epithelial CD56+ NK-cells were depleted in EC; however, no effect of obesity on NK-cell infiltration was observed. Stromal CD68+ cells were reduced in EC patients, whereas the CD163+ cells were increased. (4) **Conclusions**: Obesity and malignancy are associated with differences in immune cell presence. The alterations in immune cell infiltration seen in obese EC patients with and without diabetes suggest a complex interaction where obesity-related low-grade inflammation plays a central role.

## 1. Introduction

Endometrial carcinoma (EC) is the most common type of gynecological cancer in the western world [1,2]. Over the past 30 years, the worldwide incidence of EC has increased, with an alarming 67% increase in the Netherlands [3], and is likely multifactorial. Importantly, of all known cancer types, EC has the strongest association with obesity: every additional five body mass index (BMI) units causes a 50% increase in EC risk [4,5,6]. Underlying mechanisms for this association, i.e., how obesity stimulates the formation of malignancies, are complex. There are three main hypotheses: (1) endogenous sex-steroids production, (2) chronic hyperinsulinemia, and (3) presence of systemic inflammation [5,6,7,8]. Long-term unopposed estrogen stimulation, due to increased conversion of androgens to estrogens in adipose tissue, was long suggested as the only link between obesity and EC. Chronic hyperinsulinemia results in insulin resistance and triggers a systemic state of low-grade inflammation. Insulin resistance also increases leptin level (anti-apoptotic and pro-angiogenetic protein hormone) and decreases adiponectin level, a protein hormone which promotes cell proliferation and inhibits cell death [8,9,10,11]. Obesity-associated low-grade chronic systemic inflammation can result in a chronically activated and subsequently dysfunctional immune system [12].

In addition, obesity influences local inflammation and infiltration of immune cells in tissues and cancers, e.g., in breast and colon cancer [13,14]. Systemic hyperinsulinemia and low-grade inflammation also affect the endometrium [15], and infiltrating immune cells within stromal and epithelial compartments in EC are associated with different clinical–pathological and prognostic characteristics [16,17,18,19]. Understanding the role of obesity in EC development and its effect on systemic and local immune activity is important to elucidate EC pathogenesis and may have implications in the potential response to (immune) treatments [20,21,22] and prevention. To date, there is little information regarding the relationship between BMI and immune cell infiltration in EC. Therefore, this study explored the infiltration of immune cells in EC of obese and lean patients, compared to women with postmenopausal bleeding with benign histology. It is hypothesized, based on studies in other cancer types as well, that in obese patients there is more endometrial immune cell infiltration compared to non-obese patients [12]. However, in (advanced) stage cancer, in general, less immune cell infiltration is expected compared to healthy tissue in line with breast and colon carcinoma [14]. Therefore, we explored the local infiltration of immune cells in endometrial biopsies in patients with postmenopausal bleeding in relation to obesity and malignancy.

## 2. Materials and Methods

A retrospective cohort was created consisting of women with postmenopausal vaginal bleeding attending the gynecology outpatient clinic at Maastricht University Medical Centre (MUMC+) in 2020. Complying with Dutch guidelines, endometrial sampling was carried out (pipelle or hysteroscopic biopsy) if the endometrial thickness on transvaginal ultrasound exceeded 4 mm or in any patient presenting with recurrent blood loss [23]. From this cohort, lean patients with a BMI between 18 and 25 kg/m^2^ and obese patients with a BMI ≥ 30 kg/m^2^ were selected.

Exclusion criteria were chosen to minimize variables that could influence local immune activity and to maximize differences between groups. Exclusion of patients with BMI 25–30 kg/m^2^ was chosen to create two truly distinct BMI groups, increasing the chance of finding relevant differences in a relatively small cohort. Systemic inflammatory diseases and/or mismatch repair (MMR) deficiency can possibly alter the local immune microenvironment [24] and were therefore excluded. Non-endometrioid EC subtypes were excluded.

Patient characteristics were retrieved from electronic patient files (Table 1). Endometrial cancers were classified and graded by gyn-pathologists, according to the 2020 World Health Organization (WHO) criteria [25]. This study was approved by the Medical Ethical Committee of the MUMC+ (METC 2020-2485; Approval date: 31 March 2021) and collection, storage, and use of tissue and patient data were performed in agreement with the “Code for Proper Secondary Use of Human Tissue in the Netherlands” [26].

**Immunohistochemical staining**. Endometrial biopsies were stained for a selection of immune cells known to be related to chronic inflammation and carcinogenesis including T-lymphocytes (CD3 and CD8), Natural Killer (NK) cells (CD56), and macrophages (CD68 and CD163). Motivation for these choices is given in the Supplementary 1 [16,27,28,29,30,31]. The immunohistochemistry protocols can be found in the Supplementary 2 and Supplementary Table S1. In short, formalin-fixed, paraffin-embedded tissue (Sakura, Paraffine TEC III wax ref 4511, Torrance, CA, USA) was sectioned, deparaffinized, and rehydrated. Endogenous peroxidase activity was blocked, and antigen reactivity was restored using heat-induced epitope retrieval with buffered solutions. Double antibody staining was performed, followed by immune detection using 3,3′-diaminobenzidine (DAB) and contrast-enhancing hematoxylin stain (Sigma Aldrich: Merck KGaA, Darmstadt, Germany). The detailed immunohistochemistry protocol can be found in Supplementary 2 and Supplementary Table S1. A 3DHistech PANNORAMIC 1000 scanner (3DHISTECH, Budapest, Hungary) digitalized each slide.

**Quantification of immune cell presence using QuPath**. Quantification of immune cells in the biopsy was performed using QuPath, with manual corrections. The stepwise procedure is detailed in Supplementary 3 and Supplementary Figure S1. In short, stained slides were primarily analyzed by two authors (A.v.d.B. and E.J.) and validated by an assessor with substantial experience in endometrial pathology and/or immunohistochemistry (L.K., P.d.V.v.S., A.R., and H.W.). Blinding was applied for clinical features but impossible for pathological diagnosis. The second observer was not blinded for the primary evaluation. A measure of consistency between the first and second assessor could not be calculated due to non-blinding of the second observer. The presence of at least one mm^2^ of high-quality endometrial tissue, representative of the entire biopsy, was required for analysis. In the case of reduced availability of high-quality tissue, smaller high-quality areas were accepted after internal discussion. Regions containing both endometrial epithelium and stroma were selected as described by the International Immuno-Oncology Biomarkers Working Group [32]. An overview of samples included for analyses can be found in Supplementary Table S2. Single staining was performed for each antibody. Preferably the same area within the patient sample was evaluated for each staining. Epithelial and stromal immune cells were separately quantified, supported by QuPath. The density of infiltrating immune cells was calculated and standardized per mm^2^.

**Statistical analysis**. Differences in number and type of immune cells in endometrial biopsies were analyzed for association with clinicopathological variables, stratified by BMI and histology. The small subset of patients with a malignancy with a focus on the presence of type 2 diabetes (T2DM) was separately analyzed, in view of the known association between obesity and both EC and insulin resistance in relation to the metabolic syndrome. The Kruskal–Wallis test was used to detect differences in continuous variables (with the Mann–Whitney U test conducted as a post-hoc). Pearson Chi-Squared and Fisher’s Exact tests were applied for categorical variables. A *p*-value of ≤0.05 was considered statistically significant. Statistical analyses were performed using IBM SPSS Statistics (version 25, SPSS Inc., Chicago, IL, USA).

## 3. Results

### 3.1. Study Cohort

**Figure 1:**
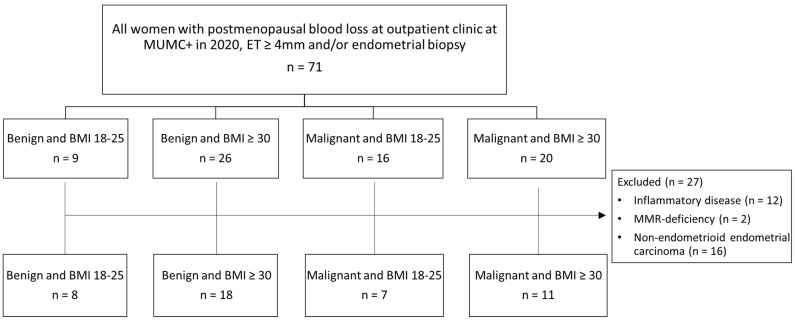
**Flowchart of patient inclusion**. Patients with postmenopausal blood loss divided by benign or malignant result on pathological examination of pipelle or biopsy and BMI groups in 2020. Because of a too-small sample size, four slender patients with a malignancy diagnosed before 2020 were added. ET, endometrial thickness; BMI, body mass index (kg/m^2^); MMR, mismatch repair. Multiple exclusion criteria are applicable to some patients.

Application of the selection criteria resulted in a cohort of 44 patients, categorized into four groups according to BMI and the etiology of postmenopausal bleeding: benign and lean (*n* = 8), benign and obese (*n* = 18), malignant and lean (*n* = 7), and malignant and obese (*n* = 11) (Figure 1). Clinicopathological characteristics are visualized in Table 1. Age was significantly higher in EC than in benign diagnosis (70 vs. 56 years, *p* = 0.001). Regarding comorbidities, the presence of cardiovascular disease did not differ between the groups. Notably within the group of obese patients, T2DM was strikingly more frequent in patients with malignancy (54.4% vs. 5.6%, *p* = 0.001). The endometrial thickness was comparable among the four groups. In EC, 17 out of 18 tumour subtypes were endometrioid, and 77.7% had early-stage disease (FIGO-stage I). All tumours were estrogen and progesterone receptor positive.

### 3.2. Immunohistochemical Staining

**Figure 2:**
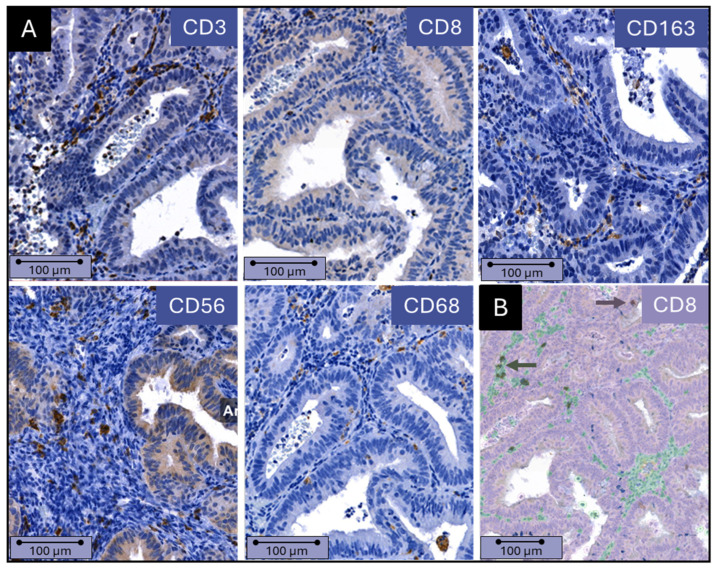
(**A**) Immune cell presence in endometrial epithelium and stroma. Example of immunohistochemical staining of CD3, CD8, CD68, CD56, and CD163 in an endometrioid adenocarcinoma. (**B**) QuPath analysis of immune cell presence and distribution. The identification of immune cells and recognition of stroma and epithelium by QuPath software (Version 0.3.2 QuPath, Northern Ireland, UK ) is shown. Stromal cells are green, and epithelial cells are purple. Immune cells located in the stroma and epithelium are dark green and dark purple, respectively (indicated by the arrows). Scale bars = 100 µm.

A total of 72% of stained samples were available for analysis (range 48–86% for the various antibodies; Supplementary Table S2) after excluding samples with areas out of focus, low-quality immunohistochemical staining, hemorrhage, or necrosis. Figure 2A shows representative images for all immune markers. Figure 2B shows an example of the QuPath overlay clearly distinguishing between epithelial cells and stromal cells, with infiltrating immune cells present in between.

#### 3.2.1. Total and Cytotoxic T-Lymphocytes (CD3 and CD8)

**Figure 3:**
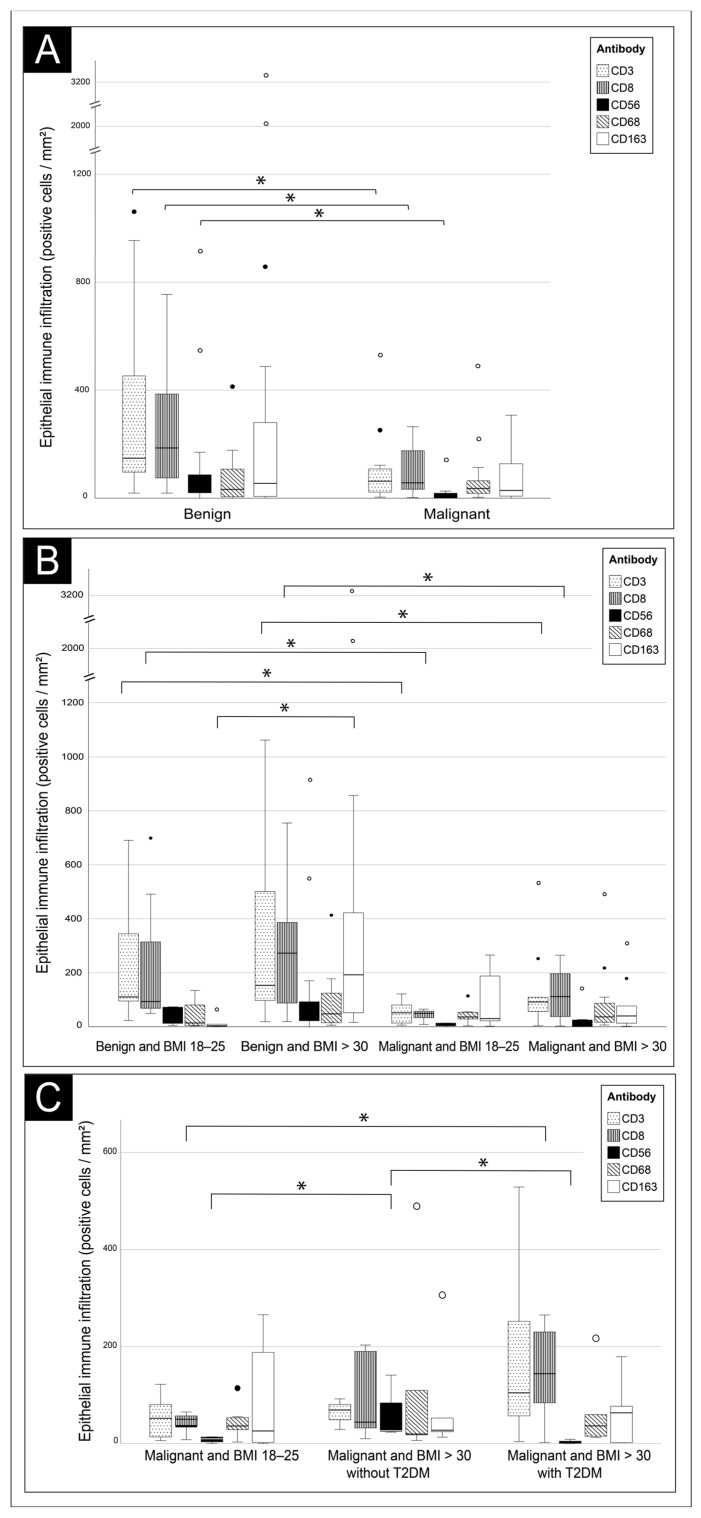
**Epithelial immune cell infiltration by diagnosis, BMI, and presence of diabetes.** Number of CD3-, CD8-, CD56-, CD68-, and CD163-positive immune cells per mm^2^ epithelium in (**A**) patients with a benign vs. malignant cause of postmenopausal blood loss, (**B**) patients with a normal BMI or obesity and benign or malignant cause of postmenopausal blood loss, and (**C**) lean vs. obese patients with a malignancy with and without the presence of diabetes type 2. BMI, body mass index (kg/m^2^); T2DM, type 2 diabetes mellitus. * *p* < 0.05. Mild (1.5 × interquartile range) and extreme outliers (3.0 × interquartile range) are represented respectively by solid and hollow dots.

Epithelial CD3+ T-cells were significantly reduced in EC compared to benign histology (63 vs. 148 cells/mm^2^ epithelium, *p* = 0.003) (Figure 3A, Supplementary Table S3). The same reduction was seen with CD8+ T-cells (57 vs. 186 cells/mm^2^ epithelium, *p* = 0.003), in both lean (50 vs. 93 cells/mm^2^ epithelium, *p* = 0.008) and obese patients (112 vs. 273 cells/mm^2^ epithelium, *p* = 0.031) (Table 2, Figure 3B). In contrast, a large increase in stromal CD3+ T-cells was seen in EC compared to benign histology, in obese patients (1095 vs. 193 cells/mm^2^ stroma, *p* < 0.001) as well as in lean patients (1617 vs. 246 cells/mm^2^ stroma, *p* = 0.042) (Table 2, Supplementary Figure S2). No difference in stromal CD8+ infiltration was seen.

Analyzing the effect of obesity on EC and benign histology, no significant effect was seen on lymphocyte infiltration. However, obese patients tended to have more intraepithelial CD8+ T-cells both in benign (93 vs. 273 cells/mm^2^ epithelium, *p* = 0.302) and EC patients (50 vs. 112 cells/mm^2^ epithelium, *p* = 0.160), though this was not significant in these small groups (Table 2, Figure 3B). Interestingly, when only obese patients with T2DM and EC are evaluated, a non-significant increase in CD3+ T-cells (105 vs. 52 cells/mm^2^ epithelium, *p* = 0.199) and a significant increase in intraepithelial CD8+ T-cells (144 vs. 50 cells/mm^2^ epithelium, *p* = 0.046) was in seen in obese diabetic patients compared to lean patients with EC, despite the small number of patients (Figure 3C, Supplementary Table S4).

To summarize, malignancy has a pronounced effect on TILs with reducing intraepithelial CD3+ and CD8+ T-cells and increasing stromal CD3+ T-cells. A possible effect of obesity is seen both in patients with benign histology and EC with more intraepithelial CD3+ and CD8+ T-cells in obese patients, especially in the presence of T2DM.

#### 3.2.2. NK-Cells (CD56)

Intraepithelial CD56+ NK-cells were significantly reduced in EC compared to benign histology (9 vs. 32 cells/mm^2^ epithelium, *p* = 0.016) (Figure 3A, Supplementary Table S3). This was observed similarly in lean (10 vs. 46 cells/mm^2^ epithelium, *p* = 0.149) and obese (9 vs. 32 cells/mm^2^ epithelium, *p* = 0.067) patients (Table 2, Supplementary Figure S2). The small subset of obese EC patients without T2DM (*n* = 5) showed a significant increase in CD56+ cells compared to those patients with T2DM (*n* = 6) (27 vs. 1 cells/mm^2^ epithelium, *p* = 0.034) and lean patients (*n* = 7) (27 vs. 10 cells/mm^2^ epithelium, *p* = 0.034) (Figure 3C, Supplementary Table S4).

To summarize, CD56+ NK-cells are depleted in the epithelium of EC in both lean and obese patients, and no clear effect of obesity on NK-cell infiltration was observed.

#### 3.2.3. Macrophages (CD68 and CD163)

No change in intraepithelial CD68+ cells was observed in EC compared to benign histology. However, stromal CD68+ cells were significantly decreased in obese EC patients vs. benign histology (115 vs. 639 cells/mm^2^ stroma, *p* = 0.004), which was to a lesser degree, also reflected in the lean group (165 vs. 420 cells/mm^2^ stroma, *p* = 0.655) (Table 2, Supplementary Figure S2). In contrast, there was a significant increase in stromal CD163+ cells in lean patients with EC vs. benign histology (472 vs. 149 cells/mm^2^ stroma, *p* = 0.037), which again was reflected in obese patients (EC vs. benign; 321 vs. 111 cells/mm^2^ stroma, *p* = 0.082) (Table 2, Supplementary Figure S2). The number of infiltrating CD68+ cells was similar in lean and obese patients. Interestingly, in obese patients with benign histology, the number of epithelial CD163+ macrophages was significantly higher than in lean patients (181 vs. 3 cells/mm^2^ epithelium, *p* = 0.014).

To summarize, stromal CD68+ cells are reduced in EC in both lean and obese patients, whereas CD163+ cells are increased. An effect of obesity is only seen in increased intraepithelial CD163+ cells in benign histology.

## 4. Discussion

Obesity and endometrial cancer are irreversibly connected, due to raised sex-steroid hormone levels in obesity and likely through obesity-related inflammatory processes [5,8]. Beyond the chronic systemic inflammation, obesity influences local inflammation and infiltration of immune cells in healthy and cancerous tissues [13,14,33]. How obesity influences immune cells infiltrating the endometrium is less known and the focus of this study.

EC had a clear effect on lymphocyte and myeloid cell infiltration. A pronounced reduction in intraepithelial CD3+ and CD8+ T-cells and CD56+ NK-cells was seen, while stromal CD3+ T-cells were increased. In the myeloid compartment, stromal CD68+ tumour-associated macrophages (TAMs)were reduced in EC, whereas CD163+ M2 were increased. Obesity on the other hand seemed to be associated with changes in immune cell infiltration with increased intraepithelial CD8+ T-cells, especially in patients with T2DM and with increased stromal CD163+ M2 macrophages.

The significantly reduced CD3+ and CD8+ T-cells in the epithelium of EC compared to benign histology are in line with the available literature. Čermáková et al. noted decreased intraepithelial CD3+ and CD8+ cytotoxic T-cells in EC [17]. An effect which became more apparent in more advanced stages of the disease, possibly related to different MHC expression [16,17,18,34]. In the EC stroma, the opposite effect was seen, with a large increase in CD3+ T-cells. This may suggest CD3+ T-helper cells gathering at the endometrial border and not being able to enter the tumour epithelium. As our study only examined the presence of immune cells rather than their functionality, we were not able to further investigate this.

It has been shown previously by Degos et al. and Antomarchi et al. that NK-cells are impoverished in the tumour microenvironment in EC [35,36] and may show an exhausted phenotype [37,38]. We confirm reduced intraepithelial CD56+ cells in EC. No effect was noted due to obesity, although it has been shown that NK-cell function is negatively affected by obesity through altered production of adipokines, Il-6 and estrogen, inhibiting their cytotoxic function, migration and proliferation [33].

TAMs affect tumour progression in various ways by cancer initiation and promotion, immune regulation, metastasis, and angiogenesis. TAMs are detected by the pan-macrophage marker CD68 and can polarize into M1 (pro-inflammatory; CD68+/CD163-) or M2 (anti-inflammatory; CD68+/CD163+) macrophages [27]. In malignant stroma, our study showed decreased CD68+ cells in obese patients and increased CD163+ cells in lean EC patients compared to their benign counterparts, which fits in a more immunosuppressive and thus pro-tumourigenic microenvironment. This was also seen in a study by Kübler et al., who also found an increased infiltration of CD163+ cells in EC using immunohistochemistry [39]. Dun et al., in contrast, demonstrated a significant increase in CD68+ TAMs in both tumour stroma and epithelium in EC compared with benign endometrium. However, this study included both non-endometrioid and high-grade EC, with increasing macrophage infiltration seen in higher FIGO stages [40]. Although in our cohort the number of infiltrating intraepithelial CD68+ cells was similar in the total group of lean and obese patients, the number of epithelial CD163+ cells was higher in obese patients. This is also underscored by Fjeldborg et al. showing a shift towards anti-inflammatory M2 macrophages in abdominal adipose tissue of obese patients [41]. This finding may indicate a polarization towards a more anti-inflammatory and dysfunctional immune environment in obese patients. Fjeldborg et al. suggested this shift towards an M2 profile may be a protective mechanism to counteract the enhanced inflammation seen in adipose tissue in obese subjects [41].

The fact that almost all T2DM patients in our cohort were found in the group of obese patients with a malignancy should be stressed. Obesity is associated with systemic low-grade inflammation [42], contributing to the development of insulin resistance, a key feature of T2DM, by disrupting insulin signaling and glucose metabolism [21,22]. As such, obesity-induced inflammation with increasing CD8+ T-cells, plays a significant role in the pathogenesis of T2DM and the metabolic syndrome [43]. These components, also in view of their proliferative and anti-apoptotic characteristics, may argue for a stronger contribution of obesity in EC development than previously thought [10,21,44,45].

Strengths of this study include the diverse panel of markers for immune cells that was used to characterize the immune environment, the additional information conveyed by the specific localization in stroma versus epithelium, and finally, the homogenous cohort of only postmenopausal women.

Limitations of this study include the small size of the patient cohort and non-blinded second assessment. QuPath application, however, strongly improved the discussion of (conflicting) results and has, as such, positively contributed to our results. Another limitation of this study is its cross-sectional design, which precludes making any causal assumptions. Longitudinal studies are needed to better understand the impact of obesity and obesity-related inflammation on the progression of malignant lesions.

Moving forward, it is crucial to understand the effect of obesity on tumour-infiltrating immune cells by further exploring the type and functionality of the involved immune cells, in larger patient cohorts. Such studies should include the measurement of systemic inflammation, especially in light of the current obesity epidemic.

## 5. Conclusions

This pilot study explored the interplay between obesity and immune cell infiltration in the context of EC and benign histology. We showed different effects of obesity and malignancy on immune cell infiltration with an increased epithelial infiltration in obesity and decreased infiltration in malignant samples. Interesting differences in immune cell presence seen in obese EC patients in relation to T2DM warrant larger studies investigating the interaction of obesity-induced inflammation. The results highlight the clinical significance of the tumour microenvironment in the pathogenic process with potential implication for the use of innovative therapies. These results on the interplay between obesity and immunology in EC need to be confirmed using larger cohorts including functional investigations.

## Figures and Tables

**Table 1:** Overview of baseline characteristics by diagnosis and BMI.

	Benign		Malignant		
	BMI 18–25	BMI ≥ 30	BMI 18–25	BMI ≥ 30	
**Included patients**	8	18	7	11	***p*-Value**
**Age** years (median; min–max)	55 (52–69)	56 ^d^ (47–71)	73 (53–76)	70 ^d^ (55–83)	0.002 ^1^*
**BMI** kg/m^2^ (median; min–max)	22.5 ^a^ (20.2–24.8)	36.0 ^a^ (30.7–49.7)	21.1 ^b^ (19.6–24.4)	39.0 ^b^ (31.6–47.9)	0.001 ^1^*
**Weight** kg (median; min–max)	62 ^a^(57–75)	94 ^a^(75–147)	58 ^b^(49–71)	100 ^b^(79–121)	0.001 ^1^*
**Comorbidities** % affected women					
**Cardiovascular disease**	3/8 (37.5%)	10/18 (55.6%)	5/7 (71.4%)	8/11 (72.7%)	0.404 ^2^
**Diabetes mellitus**	0/8 (0%)	1/18 (5.6%) ^d^	0/7 (0%) ^b^	6/11 (54.5%) ^b, d^	0.001 ^2^*
**Endometrial thickness** mm (median; min–max)	9.0 (5.3–24.0)	10.0 (6.5–28.0)	12.0 (3.8–20.0)	17.0 (2.1–33.0)	0.770 ^1^
**Pathology result**				*p*-value benign cases: 0.308 ^3^	
**Atrophy/inactive endometrium**	2/8 (25%)	10/18 (55.5%)			
**Proliferative endometrium**	1/8 (12.5%)	4/18 (22.2%)			
**Polyp**	4/8 (50%)	3/18 (16.7%)			
**Other benign result**	1/8 (12.5%)	1/18 (5.6%)		*p*-value malignant cases: 0.197 ^2^	
**Endometrioid adenocarcinoma**			6/7 (85.7%)	11/11 (100%)	
**Mucinous adenocarcinoma**			1/7 (14.3%)	0/11 (0%)	
**FIGO classification EC**					0.683 ^3^
**I**			5/7 (71.5%)	9/11 (81.8%)	
**II**			1/7 (14.3%)	1/11 (9.1%)	
**IIIa**			0/7 (0%)	1/11 (9.1%)	
**IV**			1/7 (14.3%)	0/11 (0%)	

*p*-values were calculated by ^1^ Kruskal–Wallis test, ^2^ Pearson Chi-Square, or ^3^ Chi-square test. * *p* < 0.05. Post-hoc analyses by Mann–Whitney U test or Fisher’s Exact test, and groups with significant differences (*p* < 0.05) indicated by a, b, or d. *Min*, minimum value; *max*, maximum value; *BMI*, body mass index (kg/m^2^); *EC*, endometrial carcinoma. ^0^ In two patients with malignancy, the endometrial thickness was less than 4 mm and endometrial sampling was performed only on recurrence of bleeding episodes. The bold font in the table was used to distinguish between the groups/variables and the results.

**Table 2:** Immune cell infiltration by diagnosis and BMI.

	Benign		Malignant		
	BMI 18–25	BMI ≥ 30	BMI 18–25	BMI ≥ 30	
**CD3**					***p*-Value**
**Samples analyzed**	7	17	7	9	
**Total positive cells** cells/mm^2^ (median; min–max)	268 (103–691)	229 (78–1203)	341 (22–544)	528 (139–977)	0.177
**Positive cells in the epithelium** cells/mm^2^ epithelium (median; min–max)	110 ^c^ (23–691)	153 (19–1061)	52 ^c^ (6–122)	92 (4–529)	0.016 *
**Positive cells in the stroma** cells/mm^2^ stroma (median; min–max)	246 ^c^ (109–339)	193 ^d^ (77–1492)	1617 ^c^ (73–2147)	1095 ^d^ (454–2298)	<0.001 *
**CD8**					
**Samples analyzed**	8	15	7	11	
**Total positive cells** cells/mm^2^ (median; min–max)	172 (67–667)	376 (48–1595)	102 (60–720)	140 (13–647)	0.199
**Positive cells in the epithelium** cells/mm^2^ epithelium (median; min–max)	93 ^c^ (49–699)	273 ^d^ (19–755)	50 ^c^ (8–65)	112 ^d^ (2–265)	0.006 *
**Positive cells in the stroma** cells/mm^2^ stroma (median; min–max)	158 (63–497)	396 (65–1939)	323 (186–1945)	472 (40–1275)	0.259
**CD56**					
**Samples analyzed**	4	14	4	7	
**Total positive cells** cells/mm^2^ (median; min–max)	62 ^c^ (46–81)	60 (0–319)	14 ^c^ (6–21)	46 (1–194)	0.073
**Positive cells in the epithelium** cells/mm^2^ epithelium (median; min–max)	46 (5–74)	32 (0–916)	10 (0–14)	9 (0–141)	0.098
**Positive cells in the stroma** cells/mm^2^ stroma (median; min–max)	61 (0–91)	85 (0–305)	11 (0–82)	78 (0–500)	0.270
**CD68**					
**Samples analyzed**	8	17	7	11	
**Total positive cells** cells/mm^2^ (median; min–max)	71 (13–189)	73 (5–222)	157 (67–780)	106 (26–543)	0.157
**Positive cells in the epithelium** cells/mm^2^ epithelium (median; min–max)	10 (0–135)	36 (0–413)	36 (3–114)	36 (6–489)	0.611
**Positive cells in the stroma** cells/mm^2^ stroma (median; min–max)	420 (12–1958)	639 ^d^ (81–1896)	165 (62–1204)	115 ^d^ (9–547)	0.025 *
**CD163**					
**Samples analyzed**	6	17	6	10	
**Total positive cells** cells/mm^2^ (median; min–max)	119 (59–158)	172 (0–1657)	182 (25–517)	151 (39–254)	0.333
**Positive cells in the epithelium** cells/mm^2^ epithelium (median; min–max)	3 ^a^ (0–61)	181 ^a^ (0–3263)	26 (0–266)	40 (2–307)	0.032 *
**Positive cells in the stroma** cells/mm^2^ stroma (median; min–max)	149 ^c^ (0–192)	111 (0–550)	472 ^c^ (79–1121)	321 (55–1002)	0.041 *

*p*-values between the four groups calculated by Kruskal–Wallis test. * *p* < 0.05. Post-hoc analyses by Mann–Whitney U test, and significant differences (*p* < 0.05) between two groups are indicated by a, c, or d. Min, minimum value; max, maximum value. BMI, body mass index (kg/m^2^).

## Data Availability

The raw data supporting the conclusions of this article will be made available by the authors upon request.

## Supplementary Materials

The following are available online at https://www.mdpi.com/article/10.3390/jcm13237248/s1, Supplementary 1: Selection of Immune Cells Related to Obesity and Cancer, Supplementary 2. Immunohistochemistry Protocol, Supplementary 3. Protocol Digital Image Analysis Using QuPath, Table S1. Overview of the manual immunohistochemistry procedure, showing all optimized parameters per antibody, Table S2. Overview of samples that were analyzed (numbers of analyzed epithelium and stroma/numbers of patients included), Table S3. Epithelial and stromal immune cell infiltration by diagnosis and BMI, Table S4. Epithelial and stromal immune cell infiltration in EC patients by BMI and presence of T2DM, Figure S1. Example of the baseline settings used in *positive cell detection* within QuPath, Figure S2. Epithelial and stromal immune cell infiltration.
