# Supplementary material for: Pilot Data Suggest That Obesity and Presence of Malignancy Are Associated with Altered Immune Cell Infiltration in Endometrial Biopsies
Source: J Clin Med. 2024 Nov 28;13(23):7248. doi: 10.3390/jcm13237248 (PMC11642587; doi:10.3390/jcm13237248)
Supplement: Supplementary file 1 [file jcm-13-07248-s001.zip › jcm-3252545-supplementary.pdf]

Supplementary Material

Supplementary 1. Selection of Immune Cells Related to Obesity and Cancer

T-lymphocytes play a central role in cell-mediated immunity, helping to eliminate abnormal cells. T-lymphocytes contribute to immune surveillance in the endometrium, defending against pathogens and abnormal cells [16]. Obesity is associated with chronic inflammation, and alterations in T-cell function may contribute to this inflammatory state [28,29]. CD3 is a general marker for T-lymphocytes, while CD8 identifies cytotoxic T-lymphocytes.

Natural Killer (NK) cells (in this study tested using CD56 antibodies) are part of the innate immune system and play a key role in the early defense against infected or transformed cells in both the local and systemic environment [28]. Obesity may impact NK-cell activity [29], potentially affecting their role in immune surveillance, resulting in decreased ability to target and eliminate cancer cells.

CD68 is used as a general marker for macrophages. Macrophages are phagocytic cells that digest cellular debris, pathogens, and cancer cells. Obesity is associated with an increase in macrophage infiltration, possibly contributing to (local) inflammation [29,30]. In cancer, their local presence may influence the tumour microenvironment and impact the anti-tumour immune response [36]. Generally, M1 macrophages (CD68+/CD163-) create an inflammatory response against pathogens and tumour cells, while M2 macrophages (CD163+/CD68+) typically exhibit an immune-suppressive phenotype that supports tissue repair but also aids tumour progression [27]. T2-macrophages are a subset of macrophages associated with tissue repair and anti-inflammatory responses [28,31]. In endometrial cancer, the presence of T2-macrophages may create a protumorigenic microenvironment [31]. We measured T2 macrophages using CD163.

Supplementary 2. Immunohistochemistry Protocol

Formalin-fixed, paraffin-embedded (FFPE) tissue was cut at a thickness of 5 µm using a microtome (Leica Microsystems, Machelen, Belgium) and permanently mounted. First, the paraffin-embedded slides with the endometrial tissue were deparaffinized using xylene (2×) and ethanol (VWR (Avantor), Pennsylvania, PA, USA)for 5 min and rehydrated using 100% ethanol (2×) for 2 min and 96% ethanol (1×) for 2 min. First, the endogenous peroxidase activity was blocked by incubating the tissue in a 3% hydrogen peroxide (VWR, Pennsylvania, PA, USA) in methanol solution (VWR, Pennsylvania, PA, USA) and rinsed in phosphate-buffered saline (PBS) (2×) (Thermo Scientific, Waltham, MA, USA) for 2 min. Next, heat-induced epitope retrieval (HIER) coupled with heat (95–100 °C for 20 min in a microwave) with a buffered solution (sodium citrate buffer 10 mM at pH 6 (Thermo Scientific, Waltham, MA, USA) or Tris-EDTA 50 mM at pH 9 (Tris-base: Sigma Aldrich, St. Louis, MO, USA; EDTA: Merck, Rahway, NJ, USA) was used to recover the antigen reactivity in our samples. After cooling down and rinsing the samples with PBS (2×) for 2 min, non-specific binding of other antibodies or molecules was prevented using an antibody-specific blocking solution (* BSA: Sigma Aldrich, St. Louis, MO, USA; * Goat serum: VectorLabs, Newark, CA, USA) (Table S1). After one hour, the samples were incubated overnight at 4 °C in the first antibody dilution (dilution and dilution solution of the different antibodies can be found in Table S1) using a humidified box. Non-attached antibodies were removed the next morning by rinsing the samples in PBS (3×) for 2 min. Next, the samples were incubated in Primary Antibody Amplifier Quanto for 10 min

and rinsed using PBS (3×) for 2 min. Next, the samples were incubated with the second antibody combined with horseradish peroxidase (HRP, using HRP Polymer Quanto) (Thermo Fisher Scientific, Fremont, CA, USA) for 10 min and washed with distilled water (1×) and PBS (3×) for 2 min. 3,3′-diaminobenzidine (Thermo Fisher Scientific, Fremont, CA, USA) (DAB, using a mixture of 30 μL DAB Quanto Chromogen with 1 mL of DAB Quanto Substrate) for 5 min was used for the immunodetection of our specific antigens and results in a brown precipitate that will localize the sites of antibodies that are bound by HRP. Next, a second staining method using hematoxylin (Sigma-Aldrich, St. Louis, MO, USA) for 2 min provided contrast to ensure the primary staining product could be visualized. The sections were then rinsed in running tap water for 3 min. Afterwards, the samples were dehydrated though 70% ethanol (2×) for 2 min, 96% ethanol (2×) for 2 min, 100% ethanol (2×) for 2 min, and xylene for (2×) for 5 min. The slides were cover-slipped with xylene-based rapid mounting solution (Entellan) and allowed to dry for at least 48 h before scanning. The antibody amplifier, second antibody with HRP and DAB were retrieved from the UltraVision Quanto Detection System (Thermo Scientific, Fremont, CA, USA).

Table S1. Overview of the manual immunohistochemistry procedure, showing all optimized parameters per antibody.

Antibody	Host	HIER	Blocking	First antibody dilution	Manufacturer / REF.
**CD3** (polyclonal)	Rabbit	Ci (20 min.)	1% BSA/PBS	1:500 0.1% BSA/PBS	LabNed, The Netherlands LN3100298
**CD8** (monoclonal)	Mouse	TE (20 min.)	5% GT/PBS	1:80 1% GT/PBS	LabNed, The Netherlands LN0802561
**CD56** (monoclonal)	Mouse	TE (20 min.)	1% BSA/PBS	1:250 0.1% GT/PBS	LabNed, The Netherlands LN3100365
**CD68** (monoclonal)	Mouse	Ci (20 min.)	5% GT/PBS	1:1000 1% GT/PBS	LabNed, The Netherlands LN3100381
**CD163** (monoclonal)	Mouse	Ci (20 min.)	1% BSA/PBS	1:250 0.1% BSA/PBS	LabNed, The Netherlands LN3100233

HIER: Heat-induced epitope retrieval. Ci: citric acid buffer. TE: Tris-EDTA. BSA: bovine serum albumin. GT: goat serum. PBS: phosphate-buffered saline.

Supplementary 3. Protocol Digital Image Analysis Using QuPath

Quantification of positive cells in endometrial tissue was performed using QuPath digital image analysis (version 0.3.2) with manual corrections to optimize the results. After loading, the image type was set to *Brightfield (H-DAB*). A representative area of hematoxylin and DAB was selected, and the *Estimate Stain Vectors* command was used to apply color deconvolution. First, the brush tool was used to create one or more annotations of representative regions. Then *Positive Cell Detection* was applied to distinguish the positively and negatively stained cells. Next, *Optical Density Sum* was applied using multiple detection settings to optimize the detection of positive cells. The *threshold* intensity for cell detection was used to exclude blood cells. The *Max background intensity* was used to exclude over-projected cells that showed a high intensity of hematoxylin. A *single threshold* of the DAB was adjusted to the intensity of the (background) staining. Although these optimalisation steps were individualized for each sample, due to the fact that immune cells were located in between stromal and epithelial cells, the *positive cell detection* still needed to be adjusted manually to correct for false-positive and false-negative results. An example of the default settings can be found in Figure S1. Afterwards, the *train object classifier* was used to divide the detected cells into our chosen classes: stroma and epithelium. This *object classifier* could not be reused due to the differences in staining intensity resulting from the manual staining procedure. QuPath automatically gives the results of the annotated regions. Next to the area of the annotated region, the total number of detections (cells), number of epithelial and stromal cells, and positive cells lying in between the epithelial and stromal cells were extracted from QuPath. The epithelial or stromal areas were calculated using the percentage of epithelial or stromal cells to the total number of cells.

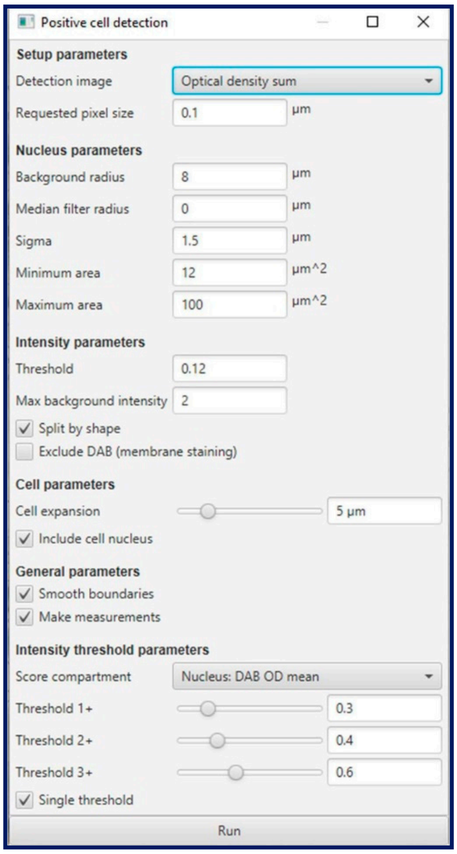

Figure S1. Example of the baseline settings used in *positive cell detection* within QuPath.

Table S2. Overview of samples that were analysed (numbers of analysed epithelium and stroma/numbers of patients included).

	CD3-CD8-CD68	CD163	CD56
Benign and BMI 18–25	88% (7/8)	71% (5/7)	57% (4/7)
Benign and BMI ≥ 30	83% (15/18)	94% (17/18)	78% (14/18)
Malignant and BMI 18–25	71% (5/7)	86% (6/7)	57% (4/7)
Malignant and BMI ≥ 30	82% (9/11)	91% (10/11)	64% (7/11)
All patients	86%	86%	66%

Table S3. Epithelial and stromal immune cell infiltration by diagnosis and BMI.

	Benign	Malignant	P-value	BMI 18-25	BMI ≥ 30	P-value
CD3						
**Samples analyzed**	24	16		14	26	
**Positive cells in the epithelium**<br>cells/mm² epithelium (median; min-max)	148<br>(19-1061)	63<br>(4-529)	**0,003 ***	95<br>(6-691)	120<br>(4-1061)	0,152
**Positive cells in the stroma**<br>cells/mm² stroma (median; min-max)	200<br>(77-1492)	1172<br>(73-2298)	**<0,001 ***	576<br>(73-2147)	474<br>(77-2298)	0,651
CD8						
**Samples analyzed**	23	18		15	26	
**Positive cells in the epithelium**<br>cells/mm² epithelium (median; min-max)	186<br>(19-755)	57<br>(2-265)	**0,003 ***	63<br>(8-699)	188<br>(2-755)	0,058
**Positive cells in the stroma**<br>cells/mm² stroma (median; min-max)	329<br>(63-1939)	418<br>(40-1945)	0,279	219<br>(63-1945)	413<br>(40-1939)	0,333
CD56						
**Samples analyzed**	18	11		8	21	
**Positive cells in the epithelium**<br>cells/mm² epithelium (median; min-max)	32<br>(0-916)	9<br>(0-141)	**0,016 ***	13<br>(0-74)	26<br>(0-916)	0,283
**Positive cells in the stroma**<br>cells/mm² stroma (median; min-max)	85<br>(0-305)	18<br>(0-501)	0,248	36<br>(0-128)	78<br>(0-501)	0,083
CD68						
**Samples analyzed**	25	18		15	28	
**Positive cells in the epithelium**<br>cells/mm² epithelium (median; min-max)	32<br>(0-413)	36<br>(3-489)	0,362	31<br>(0-135)	6<br>(0-489)	0,379
**Positive cells in the stroma**<br>cells/mm² stroma (median; min-max)	422<br>(12-1958)	130<br>(9-1204)	**0,006 ***	185<br>(12-1958)	294<br>(9-1896)	0,707
CD163						
**Samples analyzed**	23	16		12	27	
**Positive cells in the epithelium**<br>cells/mm² epithelium (median; min-max)	55<br>(0-3263)	29<br>(0-307)	0,399	7<br>(0-266)	62<br>(0-3263)	**0,021 ***

**Positive cells in the stroma** cells/mm² stroma (median; min-max)	138 (0-550)	357 (55-1121)	**0,007 ***	188 (0-1121)	173 (0-1002)	0,451

p-values calculated by Mann–Whitney U test. * $p < 0.05$. Min, minimum value; max, maximum value. BMI, body mass index (kg/m²).

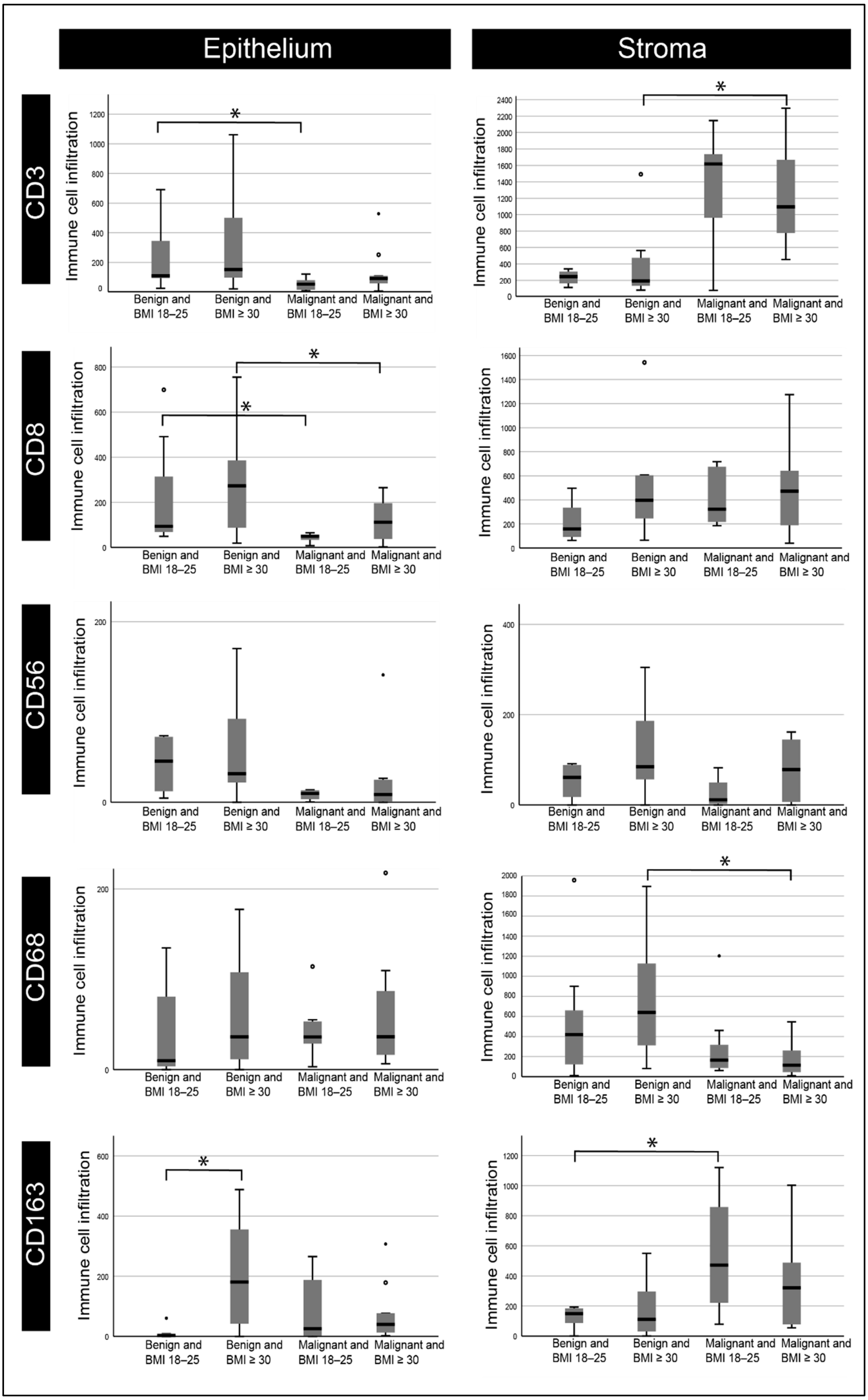

Epithelium
Stroma
CD3
CD8
CD56
CD68
CD163
Immune cell infiltration
*
Benign and BMI 18–25
Benign and BMI ≥ 30
Malignant and BMI 18–25
Malignant and BMI ≥ 30

Figure S2. Epithelial and stromal immune cell infiltration. Number of CD3-, CD8-, CD56-, CD68-, and CD163-positive immune cells per mm^2 epithelium (**left**) and stroma (**right**) in patients with a normal BMI vs. obesity split by benign or malignant cause of postmenopausal blood loss. *BMI*, body mass index (kg/m^2). Outliers are represented by dots. * $p < 0.05$. Be aware of the same interval of 200 cells but different range on the y-axis.

Table S4. Epithelial and stromal immune cell infiltration in EC patients by BMI and presence of T2DM.

Cells/mm^2 Epithelium (Median; Min-Max)	Malignant and BMI 18–25	Malignant and BMI ≥ 30 Without T2DM	Malignant and BMI ≥ 30 with T2DM
CD3	52 (6–122)	69 (29–92)	105 (4–529)
CD8	50 (8–65)	44 (10–203)	144 (2–265)
CD56	10 (0.2–14)	27 (23–141)	1 (0–9)
CD68	36 (3–114)	19 (6–489)	48 (12–218)
CD163	26 (0–266)	27 (13–307)	62 (2–179)

BMI, body mass index (kg/m^2); T2DM, type 2 diabetes mellitus.
